# Chemokine receptor 7 contributes to T- and B-cell filtering in ageing bladder, cystitis and bladder cancer

**DOI:** 10.1186/s12979-024-00432-5

**Published:** 2024-05-18

**Authors:** Jiang Zhao, Xing Luo, Chengfei Yang, Xiao Yang, Min Deng, Bishao Sun, Jingzhen Zhu, Zongming Dong, Yangcai Wang, Jia Li, Xingliang Yang, Benyi Li, Xiangwei Wang, Ji Zheng

**Affiliations:** 1 Department of Urology, The Affiliated Hospital of Guangdong Medical University, Zhanjiang, 524001 PR China; 2 Department of Urology, The Second Affiliated Hospital, Army Military Medical University, Chongqing, 400037 China; 3 Department of Urology, The University of Kansas Medical Center, Kansas City, KS 66160 USA; 4 Department of Thoracic Surgery, The Second Affiliated Hospital, Army Military Medical University, Chongqing, 400037 China; 5 Institute of Life Sciences, Chongqing Medical University, Chongqing, 400037 China

**Keywords:** Ageing bladder, Interstitial cystitis/bladder pain syndrome, Bladder urothelial carcinoma, Immune microenvironment, CCR7, Immune cell infiltration, T-cell, B-cell

## Abstract

**Background:**

Research has suggested significant correlations among ageing, immune microenvironment, inflammation and tumours. However, the relationships among ageing, immune microenvironment, cystitis and bladder urothelial carcinoma (BLCA) in the bladder have rarely been reported.

**Methods:**

Bladder single-cell and transcriptomic data from young and old mice were used for immune landscape analysis. Transcriptome, single-cell and The Cancer Genome Atlas Program datasets of BLCA and interstitial cystitis/bladder pain syndrome (IC/BPS) were used to analyse immune cell infiltration and molecular expression. Bladder tissues from mice, IC/BPS and BLCA were collected to validate the results.

**Results:**

Eight types of immune cells (macrophages, B-cells, dendritic cells, T-cells, monocytes, natural killer cells, γδ T-cells and ILC2) were identified in the bladder of mice. Aged mice bladder tissues had a significantly higher number of T-cells, γδ T-cells, ILC2 and B-cells than those in the young group (*P* < 0.05). Three types of T-cells (NK T-cells, γδ T-cells and naïve T-cells) and three types of B-cells (follicular B-cells, plasma and memory B-cells) were identified in aged mice bladder. Chemokine receptor 7 (CCR7) is highly expressed in aged bladder, IC/BPS and BLCA (*P* < 0.05). CCR7 is likely to be involved in T- and B-cell infiltration in aged bladder, IC/BPS and BLCA. Interestingly, the high CCR7 expression on BLCA cell membranes was a prognostic protective factor.

**Conclusions:**

In this study, we characterised the expression profiles of immune cells in bladder tissues of aged and young mice and demonstrated that CCR7-mediated T- and B-cell filtration contributes to the development of bladder ageing, IC/BPS and BLCA.

**Supplementary Information:**

The online version contains supplementary material available at 10.1186/s12979-024-00432-5.

## Background

Ageing, a multifactorial and natural process, is characterised by the progressive accumulation of degenerative changes involving intricate interactions among various biological and molecular mechanisms [1, 2]. Research has shown that ageing is a significant risk factor for inflammatory diseases and tumours[3–6]. Concurrently, ageing affects both innate and adaptive immune responses, thereby resulting in changes in tissue and organ immune cells and associated molecules. These changes are closely associated with tumour and inflammatory disease development and progression[6–8]. Therefore, understanding ageing-associated immune cellular and molecular changes is of great clinical value in our search for interventions for age-related diseases.

Urinary dysfunction including increased voiding frequency, urgency and urgency incontinence are common problems in ageing populations [9]. A study involving 85 female volunteers confirmed that detrusor contractility, bladder sensation and urethral pressure decreased with age [10]. Further research has confirmed that a significant linear decrease in the amount of acetylcholinesterase-positive nerve has been observed in the human bladder with increasing age, suggesting reduced parasympathetic innervation of the ageing bladder [11]. Additional animal studies have confirmed that older rats showed bladder contractile dysfunction, reduced sensitivity of pelvic nerve afferents and abnormal bladder immune cell infiltration compared with younger rats [12–14]. These studies suggest that ageing can lead to bladder dysfunction and changes in the immune microenvironment. Bacterial cystitis and interstitial cystitis/bladder pain syndrome (IC/BPS) are prevalent in older women[15–17]. Meanwhile, older patients are more susceptible to developing inflammatory bladder disease [15–17]. Further studies suggest that changes in the bladder immune microenvironment are involved in inflammatory bladder disease development and progression in older adults [18, 19]. These studies suggest that age-related molecular and cellular immune changes are involved in the pathophysiology of inflammatory bladder disease. Bladder urothelial carcinoma (BLCA) is primarily a disease of older adults, with approximately 75% of new diagnoses occurring in patients aged >65 years and approximately 45% in those aged >75 years [20, 21]. Significantly, advanced age is not only correlated with an increased risk of bladder cancer development but also fosters the emergence of aggressive tumours, which are more likely to recur and progress into invasive diseases [22, 23]. These studies suggest that old age is highly related to BLCA occurrence and development. However, in the bladder, the relationships among ageing, inflammation and cancer remain unclear. Exploring and investigating common immune cellular and molecular changes in bladder ageing, inflammation and tumours has a significant clinical value in our understanding of the pathophysiology of bladder ageing, inflammation and tumours, as well as in finding targets for intervention.

In this study, to elucidate the immune cellular and key molecular changes associated with the ageing bladder, we used single-cell RNA-seq (scRNA-seq) and transcriptome sequencing (RNA-seq) data from bladder tissues of young and aged mice. We identified chemokine receptor 7 (CCR7)-mediated T- and B-cell bladder infiltration as a significant pathway mediating bladder ageing. We further explored the expression of CCR7 in IC/BPS and BLCA and its mediated immune cell infiltration by downloading scRNA-seq and RNA-seq datasets of IC/BPS and BLCA from the Gene Expression Omnibus (GEO) and The Cancer Genome Atlas Program (TCGA) databases, respectively. This study aimed to investigate the cellular and molecular changes commonly associated with bladder ageing, inflammation and tumours and provide new insights into diagnosing and treating age-related bladder diseases.

## Results

### Mapping immune cells of the ageing bladder using scRNA-seq

Single cells from aged (18 months) and young (3 months) C57BL6J mice were obtained for analysis to explore tissue-specific immunity in ageing bladder development. A total of 10,831 cells (Supplementary Figure 1A) were filtered from aged (*n* = 5,999) and young (*n* = 4,832) mice after strict quality control (Supplementary Figure 1C). Uniform manifold approximation and projection (UMAP) analysis of the total cells identified eight types of immune cells (macrophages, B-cells, dendritic cells, T-cells, monocytes, natural killer [NK] cells, γδ T-cells and ILC2) and three types of non-immune cells (epithelial cells, myofibroblasts and neurons). Commonly characterised marker genes were selected to distinguish cell types from mice bladder tissues (Supplementary Figure 1B). Four parameters including the percentage of mitochondria, gene expression level, RNA count and percentage of ribosome were selected for quality control, and they were evenly distributed across different cell types (Supplementary Figure 1C). UMAP analysis of the cell cycle phase revealed that the percentage of G1 and G2M phases accounted mostly for immune cells compared with non-immune cells (Supplementary Figure 1D-E). Further UMAP analysis of cell types between aged and young mice bladder samples revealed some differences between the two groups (Supplementary Figure 1F). The number of T cells, γδ T cells, ILC2 and B cells were significantly increased in the bladder tissues of mice in the aged group compared to the young group. No number differences were observed in the cell types of macrophages, monocytes, NK cells, epithelial cells, myofibroblasts and neurons (Supplementary Figure 1G). To infer transcriptome regulatory networks, SCENIC packages were employed to analyse TFs between aged and young mice bladder tissues (Supplementary Figure 1H-I). TF networks among NK cells, T-cells, γδ T-cells, ILC2 and B-cells were highly different between aged mice bladder tissues and the young group. Therefore, it seems that T- and B-cell filtering played a key role in the immune microenvironment construction of the ageing bladder.

### Sub-classification of T- and B-cells in bladder tissues

**Fig. 1:**
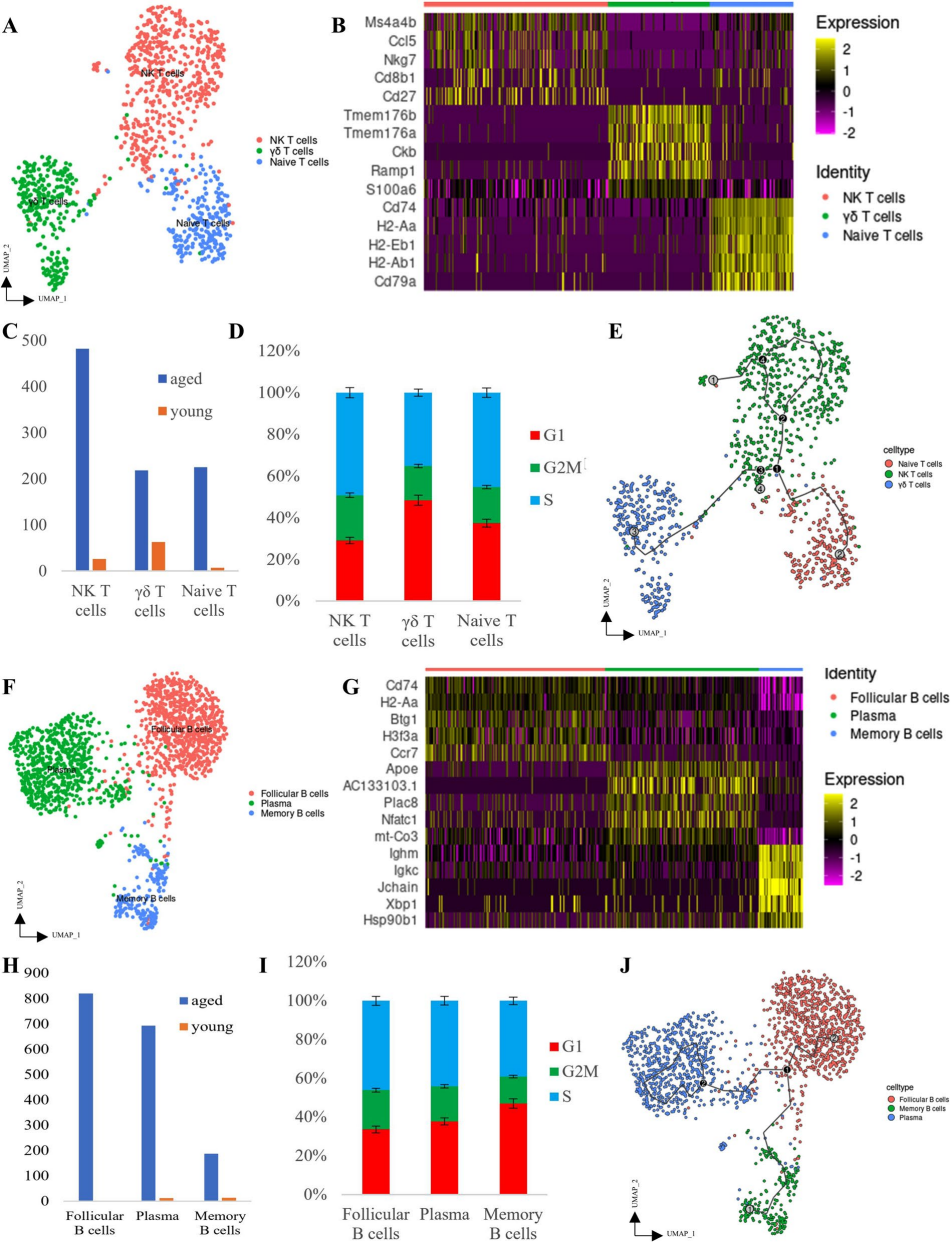
Characterisation of T- and B-cells in the aged mice bladder. **A** UMAP clustering of T-cells from mice bladder. **B** Heatmap plot of marker gene expression among the three subpopulations of T-cells. **C** Comparison of the numbers of T-cells between aged and young mice bladder tissues. **D** Comparison of the cell cycle phase of T-cells. **E** UMAP clustering of pseudo-time trajectory analysis of T-cells from mice bladder. **F** UMAP clustering of B-cells from mice bladder. **G** Heatmap plot of marker gene expression among the three subpopulations of B-cells. **H** Comparison of the numbers of B-cells between aged and young mice bladder tissues. **I** Comparison of the cell cycle phase of B-cells. **J** UMAP clustering of pseudo-time trajectory analysis of B-cells from mice bladder

To deeply understand the types of T- and B-cell filtering, sub-classification of T-cells (*n* = 720) and B-cells (*n* = 1,728) in bladder tissues was performed. Three types of T-cells (NK T-cells, γδ T-cells and naïve T-cells) were identified in the bladders of aged mice (Fig. 1A). Five significant expression markers can distinguish three types of T-cells from each other (Fig. 1B). Small amounts of γδ and naïve T-cells were observed in young mice bladder tissues (Supplementary Figure 2A and Fig. 1C). Compared with γδ T-cells, NK T-cells and naïve T-cells showed high proportions of the G2M/S phase, which denoted active proliferation (Supplementary Figure 2B and Fig. 1D). Pseudo-time trajectory analysis demonstrated developmental relationships among NK T-cells, γδ T-cells and naïve T-cells (Supplementary Figure 2C and Fig. 1E). Moreover, three types of B-cells (follicular B-cells, plasma cells and memory B-cells) were identified in the bladder of aged mice (Fig. 1F). Five significant expression markers can distinguish three types of B-cells from each other (Fig. 1G). Small amounts of plasma and follicular B-cells were present in young mice bladder tissues (Supplementary Figure 2D and Fig. 1H). Compared with memory B-cells, follicular B and plasma cells showed high proportions of the G2M/S phase, which denoted active proliferation (Supplementary Figure 2E and Fig. 1I). Pseudo-time trajectory analysis demonstrated developmental relationships among memory B-cells, follicular B-cells and plasma cells (Supplementary Figure 2F and Fig. 1J). This result suggests that both T and B cells follow the pathway of effector cell development and are activated in aged bladder tissue and may be involved in the bladder immune response.

### Comparison of signalling changes between aged and young mice bladder tissues

We performed CellChat analysis to determine the detailed signalling of T-cells and B-cell proliferation in ageing bladder tissues. These methods allow us to present a comparison framework for systematically detecting cell–cell communication across biological conditions. First, the differential number of interactions and interaction strength in the cell–cell communication network between young and aged mice bladder tissues were visualised; aged mice bladder tissues showed increased signalling compared with those of the young group (Supplementary Figure 3A). Cell–cell communication was significantly enhanced in the aged groups (Supplementary Figure 3B). The number of interactions and interaction strength among different cell types are shown in Supplementary Figure 3C. T- and B-cells demonstrated increased incoming and outgoing signalling (Supplementary Figure 3C). Second, by comparing the outgoing and incoming interaction strength in a two-dimensional space (Supplementary Figure 3D), we identified the T- and B-cell populations with significant changes in receiving signals in aged mice bladder tissues. Third, by comparing the information flow for each signalling pathway, we noted signalling pathways of MHC-I, GALECTIN and CXCL ageing-specific expression in aged mice bladder tissues. From the scatter plot, we observed that compared with the young group, MHC-I emerged as one of the major sources and targets in the aged group, whether in T or B-cells (Supplementary Figure 3G). The signalling pathways coloured red were enriched in the young group, whereas those coloured green were enriched in the aged group (Supplementary Figure 3E). Further, overall signalling, by aggregating outgoing and incoming signalling together, showed increased T- and B-cell signalling in the aged groups (Supplementary Figure 3F). Finally, we summarised the differences in the expression of five significant genes (CCR7, Cxcl13, Ccl8, Ighm and Il2rg) in T- and B-cells between aged and young mice bladder tissues (Supplementary Figure 3H).

### CCR7 was associated with T- and B-cell filtering in ageing bladder tissues

**Fig. 2:**
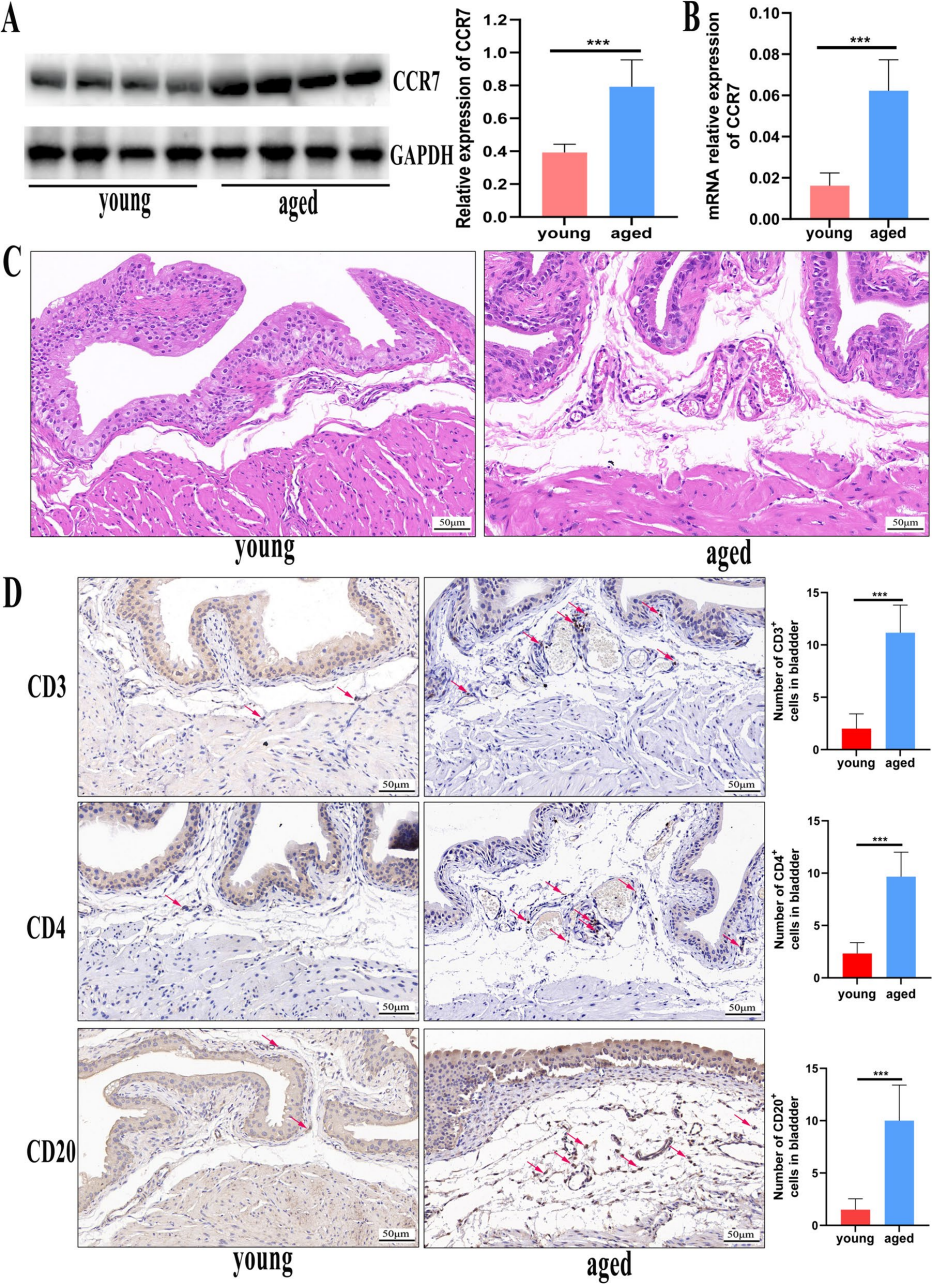
CCR7, CD3, CD4 and CD20 expression in aged and young mice bladder tissues. **A** Western blotting reveals that the CCR7 expression levels are enhanced in aged mice bladder tissues (*n* = 8) compared with those in the young normal group (*n* = 8). **B** qPCR showing that the mRNA expression levels of CCR7 are enhanced in aged mice bladder tissues (*n* = 6) compared with those in the young normal group (*n* = 6). (C) H&E-stained micrographs between aged mice bladder tissues and young normal groups. **D** Immunohistochemistry showing the CD3, CD4 and CD20 expression levels (CD3, CD4 and CD20 are mainly concentrated in the submucosal layer of the bladder, and the arrows indicate some T and B cells) between aged mice bladder tissues and young normal groups (*n* = 6).* *P* < 0.05; ** *P* < 0.01; *** *P* < 0.001; ns, not significant

To clearly understand the different pathophysiology between aged and young mice bladder tissues, we searched the GEO database (https://www.ncbi.nlm.nih.gov/geo/) and found RNA expression profiling of the bladders of ageing mice (GSE149569). Four young (3 months) and four aged (18 months) C57BL6J mice bladder tissues were adopted for RNA-seq analysis. Compared with the young group, 586 high-expression genes (fold change > 2; *P* < 0.05) and 76 low-expression genes (fold change < −2; *P* < 0.05) were noted in aged mice bladder tissues (Supplementary Figure 4A). Further analysis of the cytokine expression in eight samples revealed that most cytokines and receptors were highly expressed in aged mice bladders, including CCR7 (Supplementary Figure 4B). Moreover, 586 high-expression genes were enriched in the pathway regarding lymphocyte cell regulation, lymphocyte differentiation activation and leukocyte regulation cell–cell adhesion (Supplementary Figure 4C). More significantly, GSEA analysis indicated that the two major pathways of T-cell receptor signalling (false discovery rate [FDR] q < 0.001; normalised enrichment score [NES] = 2.34) and B-cell receptor signalling (FDR q < 0.001; NES = 2.11) from the Kyoto Encyclopedia of Genes and Genomes (KEGG) database were activated in aged mice bladder tissues (Supplementary Figure 4D–E). To perform systematic analysis of immune infiltrates across RNA-seq datasets, we used TIMER2.0 to calculate the percentage of immune infiltrates in different samples. The aged group showed a larger proportion in the ratio of B-cells and T-cell regulatory (Tregs) than the young group using TIMER algorithms (Supplementary Figure 4F). Additionally, the total type of T- and B-cells increased in the aged group compared with that in the young group using the CIBERSORT algorithm (Supplementary Figure 4G). Therefore, RNA-seq data from mice bladder confirmed that T- and B-cell filtering played an important role in ageing bladder development. Meanwhile, we observed that CCR7 expression was higher in the T- and B-cells of aged mice bladder tissues by scRNA-seq (Supplementary Figure 3H). To clearly understand CCR7 expression and immune infiltration between the bladder tissues of aged and young mice, we investigated the differences in CCR7 as well as T- and B-cells in the bladder tissues of aged and young mice. We noted that the protein (Fig. 2A) and mRNA (Fig. 2B) levels of CCR7 were significantly increased in aged mice bladder tissues compared with those of young mice. Furthermore, histological findings revealed submucosal oedema and increased inflammatory cell infiltration in the bladder tissues of aged mice compared with those of young mice (Fig. 2C). T-cell markers (CD3 and CD4) and a B-cell marker (CD20) were detected via immunohistochemistry, which revealed that the CD3, CD4 and CD20 expression levels in aged mice bladder tissues were enhanced compared with those in young mice (Fig. 2D). This result suggests that CCR7 is likely to be a significant molecule mediating T- and B-cell infiltration in the ageing bladder.

### CCR7 was associated with T- and B-cell filtering in patients with IC/BPS

**Fig. 3:**
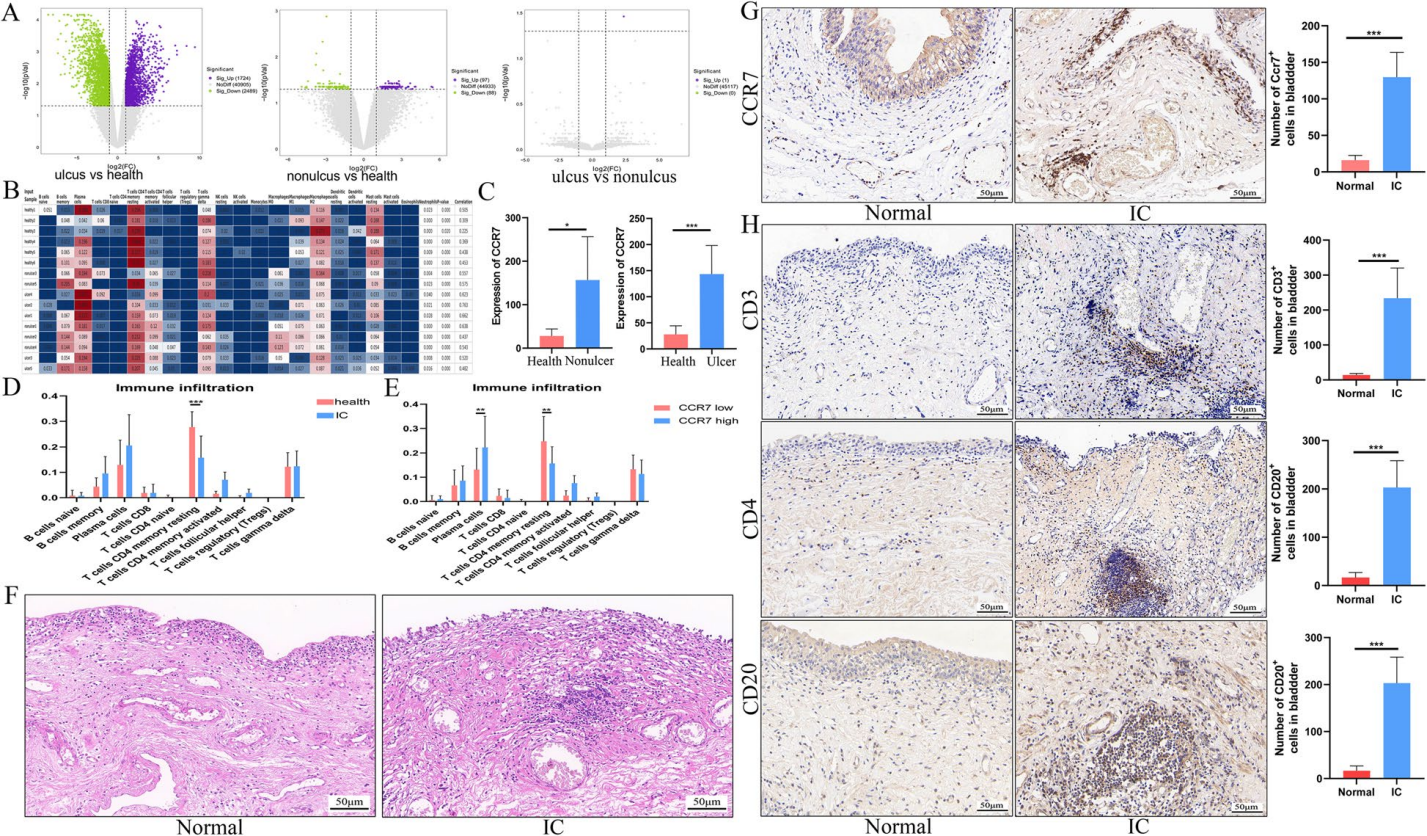
CCR7 expression and immune infiltration between IC/BPS and normal bladder tissues. **A** Volcano plot of differential genes compared between the ulcer, non-ulcer and normal groups. **B** CIBERSORT demonstrating the presence of multiple immune cell infiltrates in the IC/BPS bladder. (C) CCR7 expression is upregulated in the ulcer-IC and non-ulcer-IC groups compared with that in the control group. **D** Comparative analysis of T- and B-cell infiltration in the bladder between the normal and IC/BPS groups. **E** Comparative analysis of T- and B-cell infiltration between the low and high CCR7 expression groups. **F** Representative H&E staining for ulcerative IC. **G**–**H** Immunohistochemical staining showing the CCR7, CD3, CD4 and CD20 expression in the ulcerative IC and normal groups (*n* = 6). * *P* < 0.05; ** *P* < 0.01; *** *P* < 0.001; ns, not significant

To understand whether CCR7 mediates T- and B-cell infiltration in the context of bladder inflammation, we examined GSE11783 data and paraffin specimens of IC/BPS. As shown in Fig. 3A, the volcano plot shows differential gene expression in the normal and IC/BPS groups. As shown in Fig. 3B, a large immune cell infiltration is noted in the IC/BPS group. In this study, we observed that CCR7 was highly expressed in both ulcus and non-ulcus IC/BPS (Fig. 3C). Meanwhile, IHC further confirmed that CCR7 was highly expressed in ulcerated IC/BPS (Fig. 3G). Additionally, T- and B-cell infiltration analysis revealed the presence of predominantly CD4 memory resting T-cell infiltration in the healthy group compared with that in the IC/BPS group (Fig. 3D). Further analysis revealed that plasma and CD4 memory resting T-cell infiltration was predominantly present in the CCR7 low-expression group compared with that in the CCR7 high-expression group (Fig. 3E). Moreover, IHC showed that the CD3, CD4 and CD20 expression levels in ulcerated IC/BPS bladder tissues were enhanced compared with those in the normal group (Fig. 3I). This result suggests that in inflammatory bladder disease conditions (ulcerative IC/BPS), CCR7 expression could be closely associated with T- and B-cell infiltration.

### CCR7 gene expression was associated with T- and B-cell filtering in patients with BLCA

**Fig. 4:**
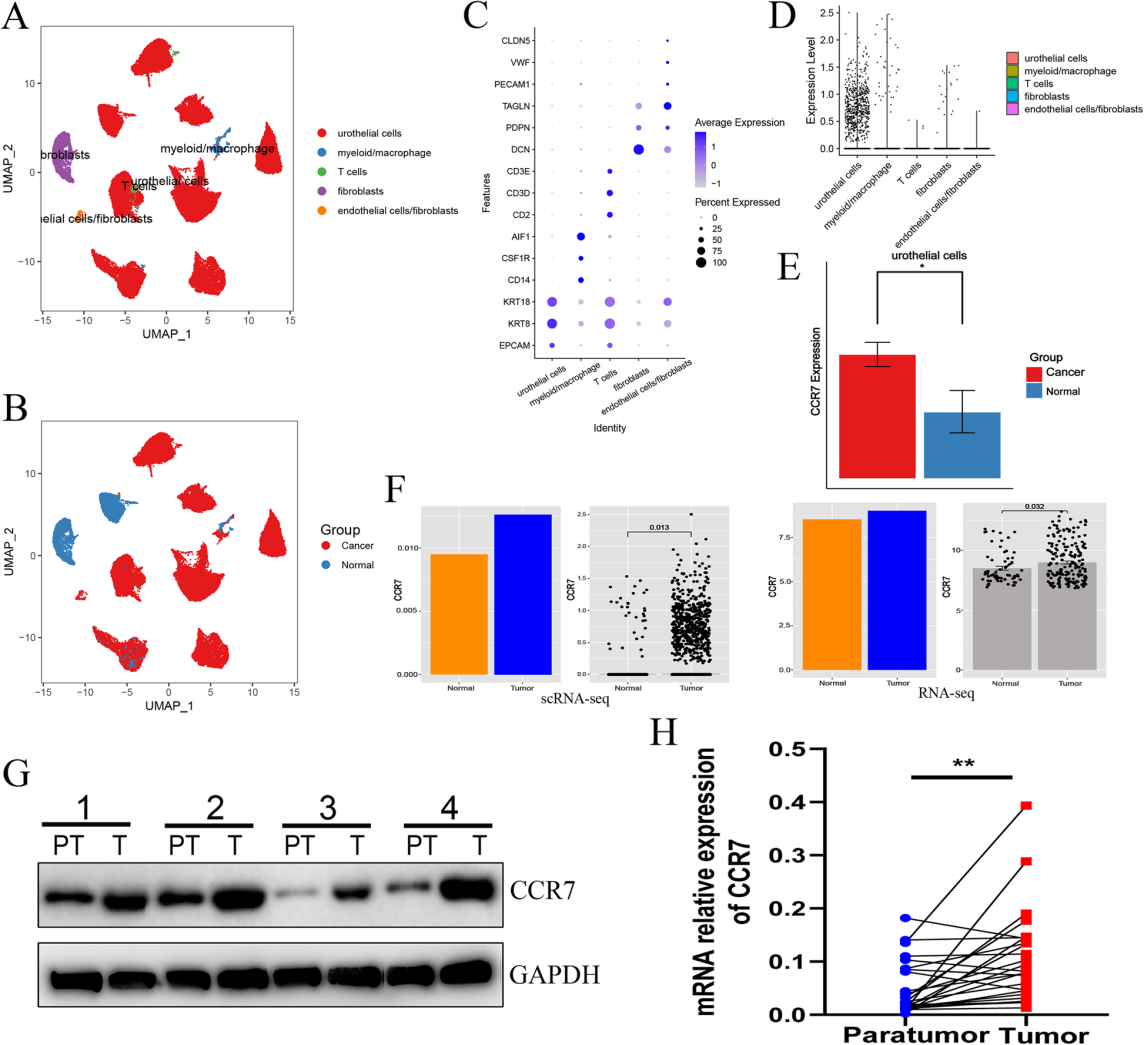
CCR7 expression in bladder cancer tissues. **A** UMAP analysis of the filtered cells from bladder cancer tissues. **B** UMAP plot of the tissue groups. **C** The dotplot of marker genes from the origianl research. **D** CCR7 expression analysis in all cell types from single-cell data. **E** The expression analysis of CCR7 in the urothelial cells of normal and bladder tumor tissues. **F** Transcriptomic data showing CCR7 expression in tumor and normal bladder tissues from scRNA-seq and RNA-seq. **G** Western blotting showing that CCR7 expression is significantly higher in bladder tumor tissues than that in paratumor tissues (*n *= 12). **H** Q-PCR showing that CCR7 mRNA expression is significantly higher in bladder tumor tissues than that in paratumor tissues. * *P* < 0.05; ** *P* < 0.01; *** *P* < 0.001; ns, not significant.

**Fig. 5:**
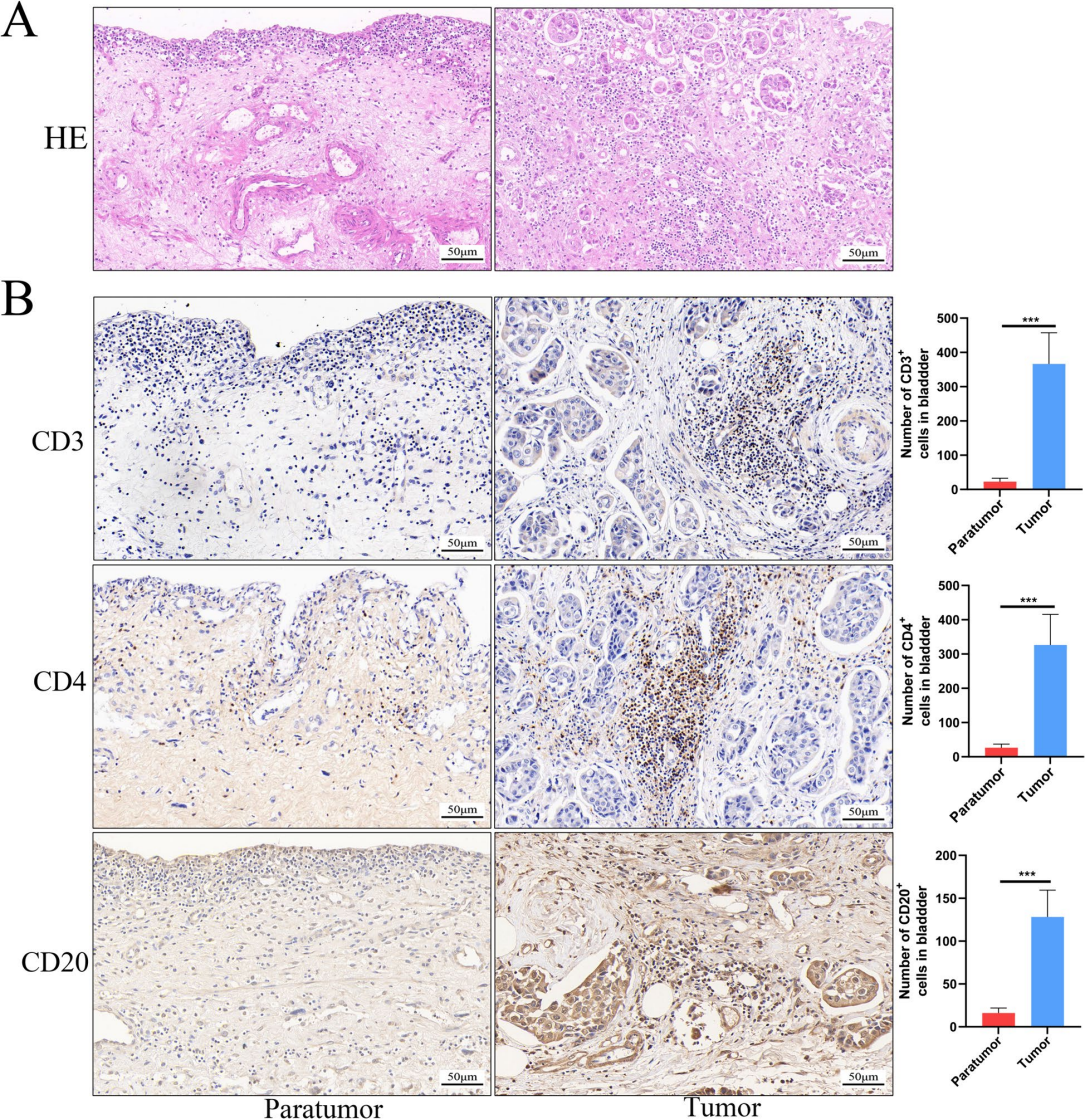
CD3, CD4 and CD20 expression in bladder cancer tumours and paracancerous tissues. **A** Representative images of H&E staining of bladder cancer tumour and paracancerous tissues. **B** Immunohistochemical staining of bladder cancer tumours and paracancerous tissues reveals changes in the CD3, CD4 and CD20 expression (*n* = 6). * *P* < 0.05; ** *P* < 0.01; *** *P* < 0.001; ns, not significant

To further explore the relationship between CCR7 and BLCA, we analysed scRNA-seq (GSE135337), RNA-seq (GSE13507) and TCGA datasets. As shown in Fig. 4A, BLCA mainly contains urothelial cells (almost tumour cells), fibroblasts, myeloid/marcrophages, T-cells and endothelial cells. The dataset consists of cells derived from tumor tissue and normal tissue. (Fig. 4B), and the cells were annoated using marker genes from the original research (Fig. 4C). As shown in Fig. 4D, CCR7 is mainly expressed on BLCA urothelial cells, and compared with the normal urothelial cells, CCR7 expressed much higher in the cancer urothelial cells (Fig. 4E) . Both scRNA-seq and RNA-seq dataset analyses(Fig 4F) suggested that tumour tissues had significantly higher CCR7 expression than normal bladder tissues. We further validated the results using clinical samples from BLCA. Through mRNA and protein expression analysis, we observed that bladder cancer tissues had a significantly higher CCR7 expression level than adjacent tissues (Fig. 4H and G). We divided BLCA samples from the TCGA database into the high- and low-expression groups according to the CCR7 expression level and analysed the correlation between CCR7 and clinical characteristics. We noted that tissues with BLCA expressed more CCR7 than paratumour tissues (Supplementary Figure 5A; *p* < 0.05). No significant difference was noted in terms of sex (Supplementary Figure 5B; male and female), tumour type (Supplementary Figure 5C; non-papillary and papillary) and pathology stages (Supplementary Figure 5D; stages i, ii, iii and iv). Additionally, we observed that the high CCR7 expression group had a higher correlation with patient age and smoking status (Supplementary Figure 5E). This result suggests that a correlation exists between CCR7 and human bladder tissue ageing. To explore whether CCR7 was correlated with T- and B-cell infiltration, we analysed the immunological profile of TCGA data for BLCA. In the ESTIMATE analysis, the high expression of the CCR7 group had higher stromal, immune and ESTIMATE scores in BLCA (Supplementary Figure 6A). The ssGSEA analysis showed that 16 immune cell subtypes (especially T- and B-cell subtypes) were highly expressed in the high CCR7 expression group (Supplementary Figure 6B-C). CIBERSORT revealed that the CCR7 expression exhibited a significant positive correlation with B-cells, Tregs and plasma cells, whereas it displayed a negative correlation with CD4 memory resting T-cells, M1 macrophages, eosinophils and resting NK cells (Supplementary Figure 6D). Finally, we validated the clinical specimens of BLCA for T-cell markers (CD3 and CD4) and B-cell markers (CD20) and noted a significant increase in T- and B-cell infiltration in BLCA tissues compared with that in paratumor tissues (*P* < 0.001; Fig. 5). These results suggest that high CCR7 expression is highly correlated with T- and B-cell infiltration in BLCA. To assess the sensitivity of CCR7 to immunotherapy in patients with BLCA, we compared the expression of common immune checkpoints between the CCR7 high and low groups. We noted that patients with high CCR7 expression levels had significantly higher levels of immune checkpoints (CD19, CTLA4 and PDCD1) (Supplementary Figure 6E). In the anti-PDL-1 therapy response, patients with high CCR7 expression had more stable disease (SD), suggesting that the effect of immunotherapy was poor (Supplementary Figure 6F). In response to anti-PD-1 therapy, we noted that patients with high CCR7 expression had significantly higher complete response and partial response than the group with low CCR7 expression, whereas they had significantly lower progressive disease than those in the low-expression group (Supplementary Figure 6G). The possible reason for this outcome could be attributed to the fact that CCR7 stimulates T- and B-cell infiltration at the tumour site, thereby leading to the killing of the tumour.

### Relationship between CCR7 expression and the prognosis of patients with BLCA

**Fig 6:**
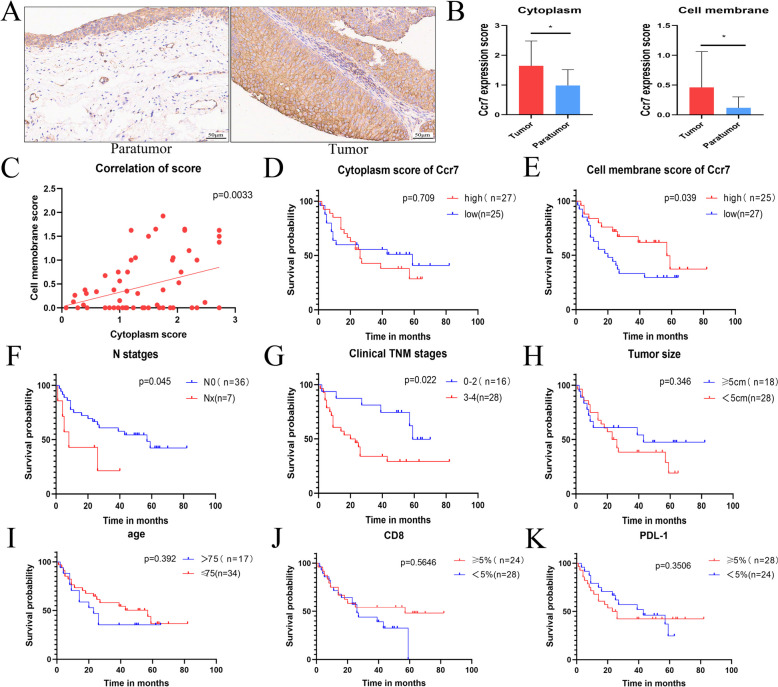
Relationship between CCR7 expression and prognosis in patients with bladder cancer. **A** Immunohistochemistry micrographs revealing CCR7 expression between paratumour and bladder tumour tissues. **B** CCR7 expression score in the cell membrane and cytoplasm between paratumour and bladder tumour tissues. **C** Correlation of CCR7 expression score between the cell membrane and cytoplasm. **D** Kaplan–Meier analysis of survival probability in patients with bladder tumour with low and high CCR7 expression cytoplasm scores. **E** Kaplan–Meier analysis of survival probability in patients with bladder tumour with low and high CCR7 expression cytoplasm scores. **F** Kaplan–Meier analysis of survival probability in patients with bladder tumour between N0 and Nx. **G** Kaplan–Meier analysis of survival probability in patients with bladder tumour between TNM 0–2 and TNM 3–4. **H** Kaplan–Meier analysis of survival probability in patients with bladder tumour between tumour sizes of <5 and ≥5 cm. **I** Kaplan–Meier analysis of survival probability in patients with bladder tumour between tumour ages of <75 and ≥75. **J** Kaplan–Meier analysis of survival probability in patients with bladder tumour between tumour CD8 < 5% and CD8 ≥ 5%. **K** Kaplan–Meier analysis of survival probability in patients with bladder tumour between tumour PDL-1 < 5% and PDL-1 ≥ 5%

We analysed TCGA data and clinical specimens to explore the relationship between CCR7 expression and prognosis in patients with BLCA. The patients were divided into high- and low-risk groups according to CCR7 expression levels. Pan-cancer analysis revealed that CCR7 was highly expressed in various tumours and that the high CCR7 expression group had better overall and disease-free survival in patients with pan-cancer (Supplementary Figure 7A and B). In BLCA, we observed that the hazard ratio (HR) value of CCR7 in the high-expression group was 0.9631 (Supplementary Figure 7C), suggesting that CCR7 was a protective factor; however, its *p* value was <0.05. Furthermore, the Kaplan–Meier survival curve showed that CCR7 alone had little effect on prognosis (Supplementary Figure 7D). Furthermore, a uniform distribution of survival states was observed among patients with high and low CCR7 expression, indicating no discernible distinction (Supplementary Figure 7E). Finally, the receiver operating characteristic curves demonstrated that the area under the curve (AUC) values for predicting 1-, 3- and 5-year prognoses were all below 0.7 (Supplementary Figure 7F), suggesting that CCR7 is not an effective prognostic marker for BLCA. Our previous findings suggest that high CCR7 expression is highly correlated with T- and B-cell infiltration and effective immunotherapy for BLCA. Moreover, research has suggested that CCR7 is a cell membrane receptor that mediates T- and B-cell migration and participates in the tumour immune response [24, 25]. Accordingly, to further elucidate the correlation between CCR7 expression in the cytoplasm and cell membrane and the prognosis of patients with BLCA, we used tissue microarrays of BLCA. As shown in Fig. 6A and B, CCR7 is highly expressed in the membrane and cytoplasm of BLCA. The correlation of CCR7 expression scores between the cell membrane and cytoplasm was statistically significant (Fig. 6C). The clinical features of the high and low CCR7 expression groups in the cytoplasm and cell membrane are shown in Supplementary Tables 1 and 2. The membranal expression of CCR7 was highly correlated with the T stage, TNM stage and CD8 positivity rate of BLCA, whereas the high cytoplasmic expression of CCR7 was highly correlated with the T and TNM stages of BLCA. Univariate regression analysis suggested that the TNM stage, CCR7 expression in the cell membrane and lymph node positivity were strongly associated with bladder cancer (Supplementary Table 3). Survival analyses showed that CCR7 expression in the cell membrane (Fig. 6E), TNM stage (Fig. 6G) and lymph node positivity (Fig. 6F) were significantly associated with patient prognosis. However, the CCR7 expression scores in the cytoplasm (Fig. 6D), tumour size (Fig. 6H), age (Fig. 6I), CD8 (Fig. 6J) and PDL-1 (Fig. 6K) did not exhibit significant associations with patient prognosis. Subsequently, multivariate Cox regression analyses showed that CCR7 expression in the cell membrane and lymph node positivity were key influences on bladder cancer prognosis (Supplementary Table 3). Interestingly, the high CCR7 expression in BLCA cell membranes is a prognostic protective factor.

## Discussion

At the population level, individuals experiencing bladder ageing, inflammation and cancer are interconnected [26]. Ageing is characterised by alterations in the production threshold of inflammatory mediators. Inflammation during ageing is considered a comprehensive model for individual ageing [27]. During the ageing process, inflammation increases and accelerates, thereby inducing the progression of ageing-related diseases [27, 28]. Additionally, ageing is the most significant risk factor for several cancer types, and it promotes tumour development and progression, whereas inflammation plays a significant role in tumour invasion, metastasis and immune escape [29, 30]. Current research has indicated that as individuals age, approximately all immune cell populations undergo changes in number and/or activity. However, these changes are typically harmful and can contribute to age-related inflammatory and tumour disease development and progression [28–32]. In the bladder, inflammatory and neoplastic diseases significantly increase with age[15–17, 20, 21]. Ageing-related alterations in immune cells and associated molecules play a role in bladder inflammation and tumour formation [18, 19, 33, 34]. The search for immune cells and molecules associated with bladder ageing, inflammation and tumours holds a significant research value for our understanding of bladder ageing and intervention in bladder ageing-related diseases.

Lower urinary tract symptoms including urinary frequency, urgency, increased nocturia and dysuria can cause significant distress in older adults. As society ages, the number of consultations for these symptoms also increases, thereby leading to greater socioeconomic burden [35, 36]. Research has indicated that bladder function declines with age, thereby resulting in lower urinary tract symptoms in older adults [35, 36]. Understanding and investigating the developmental mechanisms of bladder ageing can facilitate the effective prevention and treatment of bladder dysfunction caused by ageing. Additionally, it can provide more ideas for exploring the mechanisms of ageing. Research has suggested that the mechanisms of bladder ageing are complex, with hypoxia, oxidative stress and inflammatory responses playing a role in ageing-associated bladder dysfunction [37–39]. However, recent studies have suggested that inflammatory ageing is a significant mechanism for ageing-associated bladder dysfunction [38–41]. A previous study suggested that significant changes in the number, subpopulation and distribution of immune cells were observed in the bladders of aged mice compared with those of young mice, with an increased pro-inflammatory–associated immune cell infiltration [40]. Simultaneously, the expression of the senescence-associated secretory phenotype and pro-inflammatory markers (e.g. Cd14, Lgals3 and tnfrsf12a) increased, whereas the expression of anti-inflammatory markers (e.g. Cd9 and Cd81) decreased [41]. This indicates that the immune-inflammatory response has a significant impact on bladder ageing. In the present study, we demonstrated the cellular landscape of the immune microenvironment of the young and aged mice bladder, with a demonstration of immune cell subtypes and signalling. We noted significantly higher numbers of T-cells, γδ T-cells, ILC2 and B-cells in the bladder tissues of aged mice than in those of young mice. Infiltration of T-cells (NK T-cells, γδ T-cells and naïve T-cells) and B-cells (follicular B-cells, plasma and memory B-cells) plays a key role in immune microenvironment remodelling in the ageing bladder. During bladder ageing, a significant increase in T- and B-cell signalling and intercellular communication was observed. Additionally, T- and B-cells underwent similar developmental processes and are activated in aged bladder tissue and may be involved in the bladder immune response. Finally, single-cell, transcriptomic and molecular biology experiments confirmed that CCR7 is highly expressed in the ageing bladder and may play a crucial role in mediating T- and B-cell infiltration in the ageing bladder. This result suggests that CCR7 mediates T- and B-cell infiltration involved in the pathophysiological process of bladder ageing. Although B and T-cells significantly increased in the ageing bladder and were associated with the CCR7 receptor, further research is required to explore the underlying mechanism.

CCR7 is a G protein-coupled receptor with seven transmembrane domains. Its physiological effects are mediated by binding to its two ligands, CCL19 and CCL8, at the extracellular N terminus[42]. CCR7 is expressed in both tumour and lymphoid tissues and activates B and T lymphocyte migration towards CCR7 ligands (CCL19 and CCL21) [42]. Studies have shown that CCR7 promotes the chemotaxis of immune cells and activates inflammatory cells involved in inflammatory responses and ageing [42–44]. Abnormal changes in CCR7 expression and signalling not only trigger an immediate immune response but may also lead to a long-term inflammatory response[42]. Previous studies have shown that CCR7-positive immune cell accumulation promotes a local inflammatory response in the pancreas, thereby leading to insulin resistance [44]. Meanwhile, an increase in T-cells mediated by the CCR7–CCL19 axis is involved in the immunoinflammatory pathogenesis of dry eye [35]. Epidemiological studies have suggested that the older adult population is more prone to inflammatory bladder diseases (e.g. IC/BPS, chronic cystitis and bacterial cystitis) [15–17]. Further studies have suggested that changes in the bladder immune microenvironment are closely associated with inflammatory bladder disease development and progression in older adults [18, 19]. Previous studies have shown that abnormal T-cell, B-cell, macrophage and mast cell activation is involved in the pathophysiology of IC/BPS [18]. Previous researchers have shown that abnormalities in the CCL19–CCR7 axis are involved in the inflammatory response in interstitial cystitis [45]. In this study, we analysed IC/BPS bladder GSE11783 data and noted abnormal T-cell, B-cell, macrophage and mast cell activation in the IC/BPS bladder. Meanwhile, CCR7 was highly expressed in both ulcus and non-ulcus IC/BPS, and high CCR7 expression was closely associated with plasma and CD4 memory resting T-cell bladder infiltration. Further validation of clinical specimens from patients with ulcerative IC/BPS also revealed that CCR7 was highly expressed in these patients and that T- and B-cell infiltration was significantly increased in the IC/BPS bladder. These findings suggest that CCR7 expression in ulcerative IC/BPS is closely associated with T- and B-cell infiltration and is likely to be involved in the pathophysiology of ulcerative IC/BPS.

Advanced age is not only associated with increased levels of chronic inflammatory markers and disturbances in the gut and urinary tract microbiota but also is significantly correlated with increased BLCA incidence, morbidity and mortality [20]. In patients with BLCA, the tumours of young individuals are generally less aggressive and lower in grade than those of older individuals[22]. Additionally, the occurrence of muscle-invasive bladder cancer (MIBC) incrementally increases with advancing age within the general population[45]. Over 30% of individuals aged >85 years succumb to MIBC, in contrast to 23% of patients aged <64 years [22, 45]. The current study suggests that ageing-associated chronic inflammation and immune dysfunction are strongly associated with disease progression and poor prognosis in patients with non-MIBC and MIBC[20, 22, 45]. In BLCA, the greatest predictor of poor survival is an abnormal macrophage-to-T-cell ratio [20]. We have previously implicated CCR7-mediated T- and B-cell infiltration in the pathophysiology of bladder ageing. However, the CCR7 expression in BLCA and its impact on patient prognosis and its relationship with immune cell infiltration have not been elucidated. Previous studies have demonstrated that CCR7 is highly expressed in BLCA and that CCL21/CCR7 may promote BLCA development and metastasis [22, 45]. In this study, we observed that CCR7 was highly expressed in the cell membrane and cytoplasm of patients with BLCA, and high CCR7 expression was highly correlated with the age of patients with BLCA. This result further supports the involvement of CCR7 in the pathophysiology of bladder ageing in humans and mice. Pan-cancer analysis revealed that CCR7 was highly expressed in various tumours and that the high CCR7 expression group had better overall and disease-free survival in pan-cancer. However, bioinformatic analyses suggested that CCR7 had little or no effect on the survival prognosis of patients with BLCA. However, CCR7 is a cell membrane receptor that mediates T- and B-cell migration and is involved in the tumour immune response [24, 25]. Hence, to investigate the association between patient prognosis and CCR7 expression in the cytoplasm and cell membrane, we utilised a tissue microarray comprising 55 patients with BLCA. Using multivariate Cox regression analysis, we observed that the survival prognosis of BLCA was strongly associated with cell membrane CCR7 expression and lymph node positivity. Interestingly, we noted that high cell membrane CCR7 expression was a prognostic protective factor in BLCA, which is consistent with the results of pan-cancer research. Further analysis showed a strong correlation between high CCR7 expression and T- and B-cell infiltration in BLCA. Meanwhile, we observed that high CCR7 expression improves the response to anti-PD-1 therapy in patients with BLCA. A possible reason for this finding is CCR7 chemotactic T- and B-cell infiltration at the tumour site, and the infiltrating T- and B-cells are involved in the immune tumour killing associated with BLCA.

In the present study, we found that CCR7 may mediate T-cell and B-cell filtration and thus be involved in bladder ageing, IC/BPS and BLCA processes. However, our study still has the following limitations. First, the dataset and sample included in this study are small, and the findings have some limitations. Second, our study lacks single-cell sequencing and comparative studies of bladder tissues from young and older adults. These findings do not fully represent the changes in human bladder tissue ageing. To understand the pathophysiology of bladder ageing, further human specimen studies are needed. Third, owing to the difficulty of obtaining IC/BPS specimens in China, only specimens from patients with ulcerative IC/BPS were included in this study, and non-ulcerative IC/BPS specimens were lacking. Therefore, the results of the study were limited to patients with ulcerative IC/BPS. To understand the relationship between bladder ageing and inflammation, further specimen collection and validation efforts are needed. Fourth, the findings have some limitations owing to the high degree of tumour heterogeneity in BLCA and the small sample of patients with BLCA included in this study. Future studies with large sample sizes will require further validation of the findings. Fifth, the single-cell data of bladder tissues in this study were obtained from public databases and lacked bladder smooth muscle cells, and future single-cell sequencing studies of whole bladder tissues need to further explore the microenvironment of bladder tissues. Sixthly, the specific molecular mechanisms by which CCR7 mediates T- and B-cell infiltration are not explored in this study, and to elucidate this issue, further experimental studies are required.

## Conclusions

We identified the expression profiles of different cells in bladder tissues from aged and young mice and confirmed that T- and B-cell filtering contributed to aging, cystitis, and BLCA development. The signalling receptor CCR7 provides deep insight into the molecular mechanisms of ageing bladder and facilitates the discovery of novel biomarkers for cystitis and BLCA treatment. The identification of CCR7 as a potential biomarker for these processes opens up new avenues for research and therapeutic intervention. However, to confirm the role of CCR7 in these processes and explore its potential as a therapeutic target, further studies are needed.

## Materials and methods

### Experimental datasets and animal sample acquisition

The scRNA-seq (GSE149564) and RNA-seq (GSE149569) data from young (3 months) and aged (18 months) mice were obtained from the GEO database (https://www.ncbi.nlm.nih.gov/geo/) and were used for analyses in this study. BLCA scRNA-seq (GSE135337) and RNA-seq (GSE13507) were obtained from the GEO database and used for analysis. The GSE135337 dataset was annotated to different cell types based on markers provided in the literature [46]. Meanwhile, the high- and low-expression groups were classified according to the median CCR7 expression. The GSE11783 data for IC/BPS were obtained from the GEO database, and the analysis method is consistent with that of our previous report [47]. Young (3 months) and aged (18 months) C57BL/6 mice were purchased from the Army Military Medical University Animal Center. Mice were kept at 20°C–25°C in a standard environment for the same length of day and night with free access to food and water. This study was approved by the Ethics Committee of China Army Medical University, China (approval no.: 2024-YD-021-01) and was performed in accordance with the Animal Welfare Guidelines and the Declaration of Helsinki.

### scRNA-seq data processing

scRNA-seq data analysis and plot were mainly performed using the R package Seurat, which is designed for quality control, analysis and exploration of scRNA-seq data [48]. Additionally, Seurat was used to identify and interpret sources of heterogeneity from scRNA-seq measurements. The transcription factors (TFs) analysis was performed using the single-cell regulatory network inference and clustering (SCENIC) workflow, which utilises three separate packages including GENIE3 or GRNBoost2, RcisTarget and AUCell [49]. Co-expression modules between transcription factors and candidate target genes were inferred based on co-expression using GENIE3 or GRNBoost2. Cisregulatory motif analysis was performed for each co-expression module using RcisTarget. Scoring of each regulon activity in each cell using the AUCell algorithm. TIMER2.0 is a comprehensive resource for the systematic analysis of immune infiltrates across diverse cancer types [50]. This version of the web server provides immune infiltrate abundances estimated by multiple immune deconvolution methods and allows users to dynamically generate high-quality figures to comprehensively explore tumour immunological, clinical and genomic features. Immune infiltration estimations for user-provided expression profiles by TIMER2.0, CIBERSORT, quanTIseq, xCell, MCP-counter and EPIC algorithms (http://timer.cistrome.org/). To explore ligand–receptor interaction and cell–cell communications, which allows systematically detecting dysregulated cell–cell communication across biological conditions, CellChat R packages were employed [51].

### TCGA data analysis

BLCA data were downloaded from TCGA database. The data shown here are in part based on data generated by the TCGA Research Network: https://www.cancer.gov/tcga. Clinical, RNA-seq and pathological data are available in the Genomic Data Commons Data Portal. Matched TCGA patient identifiers allow the study of clustering and correlations between cluster type and clinical characteristics. Data preprocessing was performed as follows: (1) TCGA RNA-seq data with fragments per kilobase million (FPKM) standardise was used for further analysis; (2) 408 tumour tissues and 19 matched paratumour tissues from 408 patients with BLCA; (3) CCR7 gene expression FPKM data were selected for analysis in all tumour samples and matched paracancerous tissues; and (4) excluded samples without clinical information. In this study, we categorised the high- and low-expression groups according to the median CCR7 expression. To calculate the immune score of patients with TCGA, the estimation algorithm was used. Single-set GSEA (ssGSEA) and CIBERSORT algorithms were used to calculate the immune microenvironment of patients with TCGA, and the correlation between CCR7 and immune cells was calculated. The correlation between CCR7 and programmed cell death ligand 1 (PDL-1) immunotherapy efficacy was evaluated using the R package IMvigor210CoreBiologies to assess differences in immune checkpoint expression in the high-altitude group. The GEO dataset GSE91061 was used to assess the association of CCR7 with PDL-1 treatment [52]. Pan-cancer survival of CCR7 is available on the GEPIA website (http://gepia.cancer-pku.cn/). Expression difference analysis in TIMER2.0 website (http://timer.cistrome.org/).

### Clinical sample collection

Twelve paired BLCA carcinomas and paracancerous tissues were collected from the Second Affiliated Hospital of Army Military Medical University and the Affiliated Hospital of Guangdong Medical University from January 2022 to June 2023 and were used for western blot studies. Bladder cancer tissues were obtained from patients undergoing radical cystectomy. All tissues were diagnosed by more than two pathologists following routine haematoxylin and eosin (H&E) staining. For clinical evaluation, human BLCA tissue microarrays (HLugA180Su08, HLugA180Su04, ShGnghGi Outdo Biotech Company) containing 47 tumours and 16 paired cancer and paracancerous tissues were used. The patient’s age, tumour grading, tumour staging, PDL-1 expression and other clinical data were collected for analysis. Tissue microarrays were scanned on whole slides using Leica's Aperio XT scanner. Tissue microarray immunohistochemistry results were scored diagnostically by an experienced pathologist who first read the type of positive staining (nuclear, cytoplasmic and membranous). In this experiment, positive staining was seen in the cell membrane and cytoplasm, and the percentage of cells positive (0-100%) for membrane and cytoplasm and the intensity of positivity on a scale of 0-3, where 0 is negative, 1 and 2 are weakly positive, and 3 is strongly positive. The total score was calculated as the product of the staining intensity score and the staining positivity score. Survival analysis groupings were divided into high and low expression groups according to the median total score. Paraffin sections of normal bladder tissue from six patients with cancer and six bladder tissue samples from patients with ulcerative IC/BPS were collected for the study. The standard for collecting normal bladder tissue is 5 cm away from the cancerous tissue. This study was approved by the Ethics Committee of the Second Affiliated Hospital of Army Military Medical University and was conducted in accordance with the Declaration of Helsinki. The study participants provided the explicit consent of both their family members and themselves and signed an informed consent form.

### H&E and immunohistochemical (IHC) staining

Human and mouse bladder paraffin specimens were collected for H&E staining and IHC analysis. H&E staining was performed following our previously reported method [47]. The IHC staining and scoring methods were consistent with our previously reported methods and procedures [53, 54]. The antibody concentrations for IHC were as follows: anti-CCR7 recombinant rabbit monoclonal antibody (1:10,000, SR36-04, Huabio, China), anti-CD3 rabbit monoclonal antibody (1:200, GB13014-50, Servicebio, China), anti-CD4 rabbit polyclonal antibody (1:100, GB11064-100, Servicebio, China) and anti-CD20 rabbit polyclonal antibody (1:200, GB11540-100, Servicebio, China). A universal reagent kit mouse mouse/rabbit polymer detection system (PV-6000, Zhongshan Inc., China) was used for antibody detection.

### Western blot analysis

Western blotting experimental methods and procedures were consistent with those previously reported [55, 56]. For electrophoresis, 50-μg protein was used. The following were the antibody concentrations for western blotting: anti-CCR7 recombinant rabbit monoclonal antibody (1:10,000, SR36-04, Huabio, China) and mouse monoclonal anti-GAPDH (1:1,000, 60004-1-Ig, Proteintech, China).

### Quantitative real-time polymerase chain reaction (PCR)

Cancerous and paracancerous tissues from 25 pairs with BLCA were collected from the Second Affiliated Hospital of Army Military Medical University and the Affiliated Hospital of Guangdong Medical from January 2022 to June 2023. Quantitative real-time PCR was performed as previously reported [57]. The primer sequences for CCR7 are provided in Supplementary Table 4.

### Statistical analysis

Categorical variables are presented as numbers and proportions, whereas continuous variables are presented as means with standard deviations (SDs). Categorical variables were compared using analysis of variance (ANOVA) or chi square test, whereas continuous variables was compared using Student’s t-test (comparison of data with normal distribution), Mann–Whitney test (comparison ranks) or Kolmogorov–Smirnov test (comparison of cumulative distributions) depending on the results of the normality distribution test. To estimate the effect of each predictor on overall survival and recurrence-free survival (RFS), univariate Cox proportional risk models were used. To identify the independent predictors of RCOS and RFS, multivariate Cox proportional risk models were used. The gene set enrichment analysis (GSEA) platform was used for pathway analysis. One-way ANOVA with Kruskal–Wallis statistical test was performed using GraphPad Prism version 6.04 for Windows (GraphPad Software, La Jolla, CA, USA, www.graphpad.com). R software (https://www.r-project.org/) was employed for statistical analysis. *P* < 0.05 was considered statistically significant (NS: not significant, *P < 0.05, **P < 0.01, ***P < 0.001).

### Supplementary Information

Supplementary Material 1: Supplementary Figure 1. 10× single-cell RNA-seq analysis of ageing bladder tissues in mice. (A) UMAP analysis of the filtered cells (*n* = 10,831) from mice bladder tissues. (B) Dotplot showing scaled expression levels for marker genes of each cell type in mice bladder. (C) Violin plots showing average expression levels of quality control parameters for different cell types in mice bladder. (D) UMAP analysis of cell cycle scores in different cell types. (E) Comparison of cell cycle scores of each cell type from mice bladder tissues. (F) UMAP analysis of the cell type distribution between aged and young mice bladder tissues. (G) Comparison of the numbers of each cell type between aged and young mice bladder tissues. (H) SCENIC analysis of TFs in young mice bladder tissues. (I) SCENIC analysis of TFs in aged mice bladder tissues.* *P* < 0.05; ** *P* < 0.01;***P < 0.001; ns, not significant.

Supplementary Material 2: Supplementary Figure 2. Sub-classification of scRNA-seq data from T- and B-cells. (A) UMAP analysis of T-cells from aged and young mice bladder tissues. (B) UMAP analysis of the cell cycle phase in T-cells. (C) Pseudo-time trajectory analysis in the sub-classification of T-cells in mice bladder. (D) UMAP analysis of B-cells from aged and young mice bladder tissues. (E) UMAP analysis of the cell cycle phase in B-cells. (F) Pseudo-time trajectory analysis in the sub-classification of B-cells in mice bladder.

Supplementary Material 3: Supplementary Figure 3. Cell–cell interaction of bladder cells between aged and young mice. (A) Comparison of the number of interactions between aged and young mice bladder tissues. (B) Histogram analysis of the number of inferred interactions and strength between aged and young mice bladder tissues. (C) Comparison of the number of interactions and strength in different bladder cell types between aged and young mice. (D) Comparison of outgoing and incoming interaction strength between aged and young mice bladder cells. (E) Comparison of signalling flow within the inferred networks between aged and young mice bladder cells. (F) Comparison of overall signalling patterns within the inferred networks between aged and young mice bladder cells. (G) Comparison of signalling changes in B and T-cells between aged and young mice. (H) Violin plots showing the average expression levels of Cxcl13, CCR7, Ccl18, Ighm and Il2rg between aged and young mice bladder cells.

Supplementary Material 4: Supplementary Figure 4. mRNA-seq analysis of gene expression in aged and young mice bladder tissues. (A) Volcano plot showing 586 high-expression genes (red colour) and 76 low-expression genes (blue colour) in aged mice bladder tissues (*n* = 4) compared with those in the young group (*n* = 4). (B) Heatmap plot of high and low cytokine expressions between aged and young mice bladder tissues. (C) Gene Ontology enrichment analysis of high-expression genes (*n* = 586) in aged mice bladder tissues. (D) GSEA analysis showing the KEGG pathway of T-cell receptor changes in aged mice bladder tissues compared with those in the young normal group. (E) GSEA analysis showing the KEGG pathway of B-cell receptor changes in aged mice bladder tissues compared with those in the young normal group. (F) Comparison of immune cell filtering between aged and young mice bladder tissues using the TIMER2.0 algorithm. (G) Comparison of immune cell filtering between aged and young mice bladder tissues using the CIBERSORT algorithm.

Supplementary Material 5: Supplementary Figure 5. Comparative analysis of the expression in the TCGA dataset with clinically relevant tumour data. (A) Histogram plot showing the difference in CCR7 expression between paratumour and tumour bladder tissues. (B) Histogram plot showing the difference in CCR7 expression between bladder tissues of male and female patients. (C) Histogram plot showing the difference in CCR7 expression between different types of bladder tumour tissues.(D) Histogram plot showing the difference in CCR7 expression among different stages of bladder tumour tissues. (E) Correlation analysis of CCR7 high- and low-expression groups with clinical features in BLCA samples from the TCGA database.

Supplementary Material 6: Supplementary Figure 6. Correlation between CCR7 expression and immune infiltration and immunotherapy in bladder cancer. (A) Analysis of stromal, immune and ESTIMATE scores in the high and low CCR7 expression groups of bladder cancer. (B) ssGSEA analysis showing the distribution of immune cells in the high and low CCR7 expression groups. (C) Correlation analysis between CCR7 expression and T- and B-cell infiltration. (D) CIBERSORT analysis showing immune cell distribution. (E) Differential expression of immune checkpoint molecules between the high and low CCR7 expression groups. (F and G) Anti-PD-L1 and anti-PD-1 therapy response in the high and low CCR7 expression groups. * *P* < 0.05;** *P* < 0.01; *** *P* < 0.001; ns, not significant.(TIF 23629 KB)

Supplementary Material 7: Supplementary Figure 7. Prognostic analysis of CCR7 expression and bladder cancer. (A) Expression analysis of CCR7 in pan-cancer. (B) CCR7 expression in pan-cancer analysed with overall tumour survival and disease-free survival. (C) Forest plot analysis of the HR of CCR7 in patients with bladder tumour. (D) Kaplan–Meier survival curves of time for patients with high and low CCR7 expression in bladder cancer. (E) Survival state distribution in patients with bladder cancer between high and low CCR7 expression. (F) AUC showing the efficiency of CCR7 in predicting outcomes in patients with bladder cancer.

Supplementary Material 8: Supplementary Table 1. Clinical features of the high and low CCR7 expression groups in the cell membrane.

Supplementary Material 9: Supplementary Table 2. Clinical features of the high and low CCR7 expression groups in the cytoplasm.

Supplementary Material 10: Supplementary Table 3. Cox regression analysis.

Supplementary Material 11: Supplementary Table 4. Quantitative real-time PCR.

## Data Availability

Analyses and visualisations of the scRNA-seq, RNA-seq and TCGA datasets from this study are available in the GEO database. Other data from this study are available from the corresponding author.
