# Supplementary figures and images for: Chemokine receptor 7 contributes to T- and B-cell filtering in ageing bladder, cystitis and bladder cancer
Source: Immun Ageing. 2024 May 18;21:33. doi: 10.1186/s12979-024-00432-5 (PMC11102276; doi:10.1186/s12979-024-00432-5)

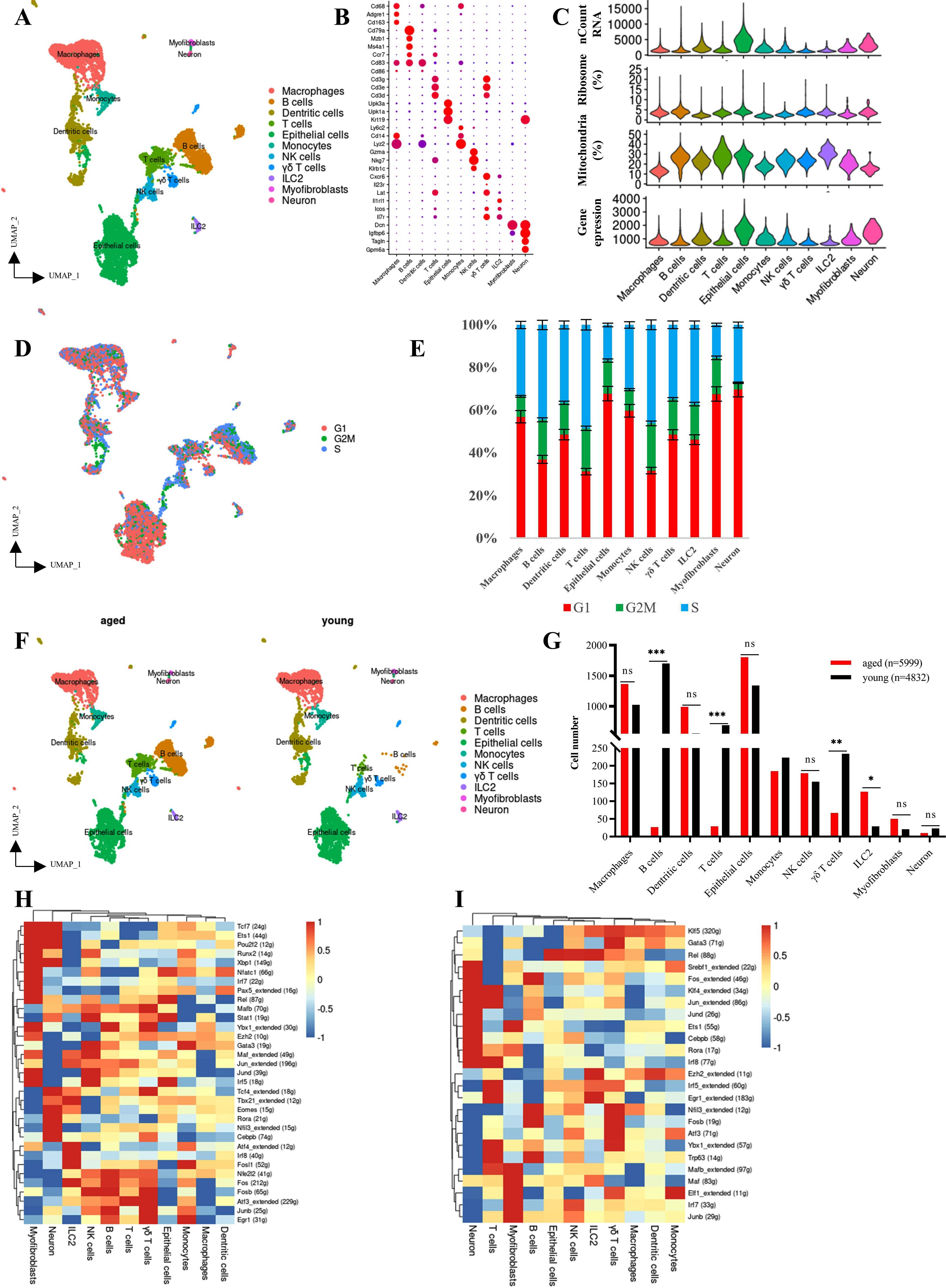

Supplement: Supplementary file 1 — Supplementary Material 1: Supplementary Figure 1. 10× single-cell RNA-seq analysis of ageing bladder tissues in mice. (A) UMAP analysis of the filtered cells (n = 10,831) from mice bladder tissues. (B) Dotplot showing scaled expression levels for marker genes of each cell type in mice bladder. (C) Violin plots showing average expression levels of quality control parameters for different cell types in mice bladder. (D) UMAP analysis of cell cycle scores in different cell types. (E) Comparison of cell cycle scores of each cell type from mice bladder tissues. (F) UMAP analysis of the cell type distribution between aged and young mice bladder tissues. (G) Comparison of the numbers of each cell type between aged and young mice bladder tissues. (H) SCENIC analysis of TFs in young mice bladder tissues. (I) SCENIC analysis of TFs in aged mice bladder tissues.* P < 0.05; ** P < 0.01;***P < 0.001; ns, not significant. [file 12979_2024_432_MOESM1_ESM.tif]

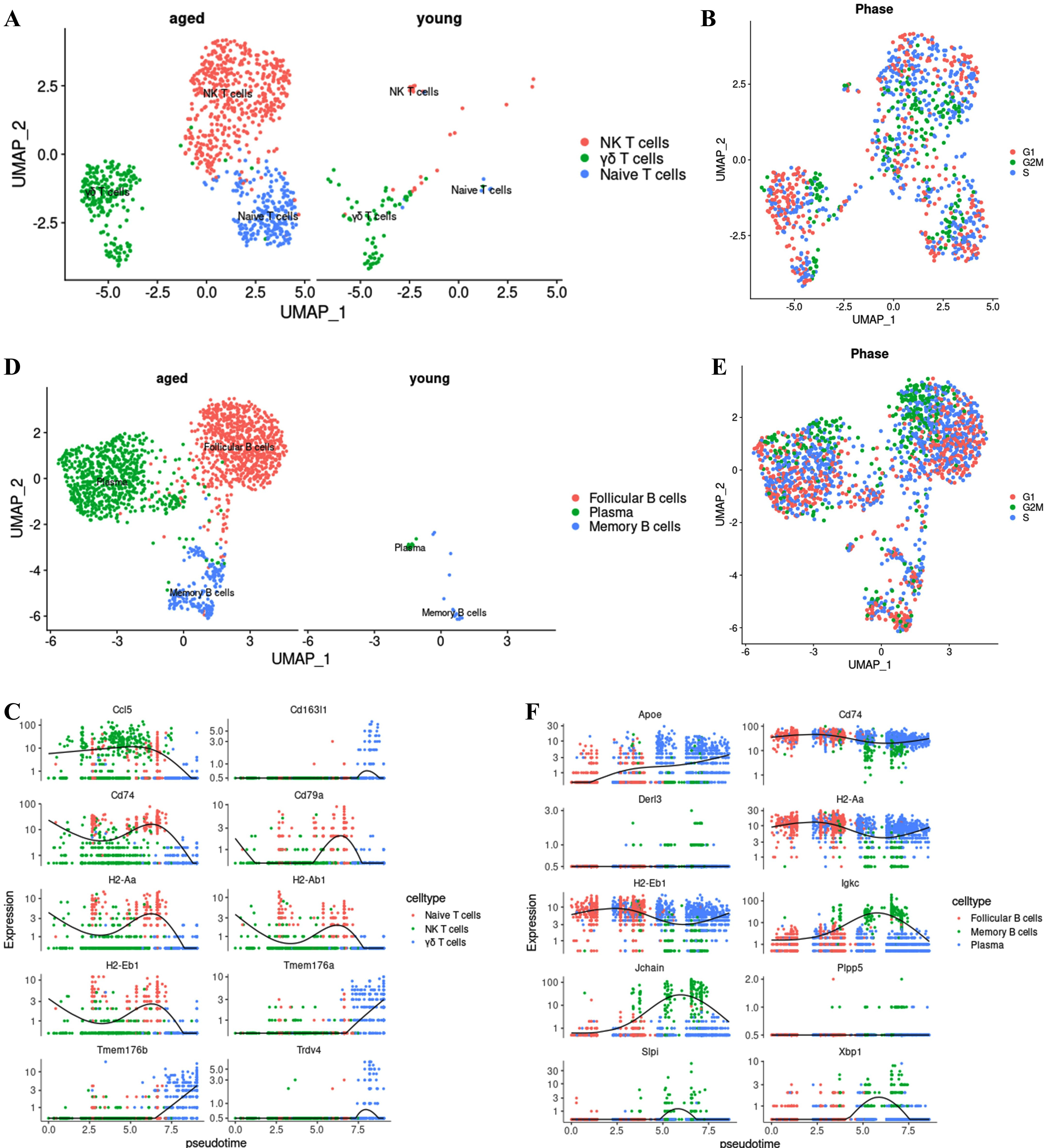

Supplement: Supplementary file 2 — Supplementary Material 2: Supplementary Figure 2. Sub-classification of scRNA-seq data from T- and B-cells. (A) UMAP analysis of T-cells from aged and young mice bladder tissues. (B) UMAP analysis of the cell cycle phase in T-cells. (C) Pseudo-time trajectory analysis in the sub-classification of T-cells in mice bladder. (D) UMAP analysis of B-cells from aged and young mice bladder tissues. (E) UMAP analysis of the cell cycle phase in B-cells. (F) Pseudo-time trajectory analysis in the sub-classification of B-cells in mice bladder. [file 12979_2024_432_MOESM2_ESM.tif]

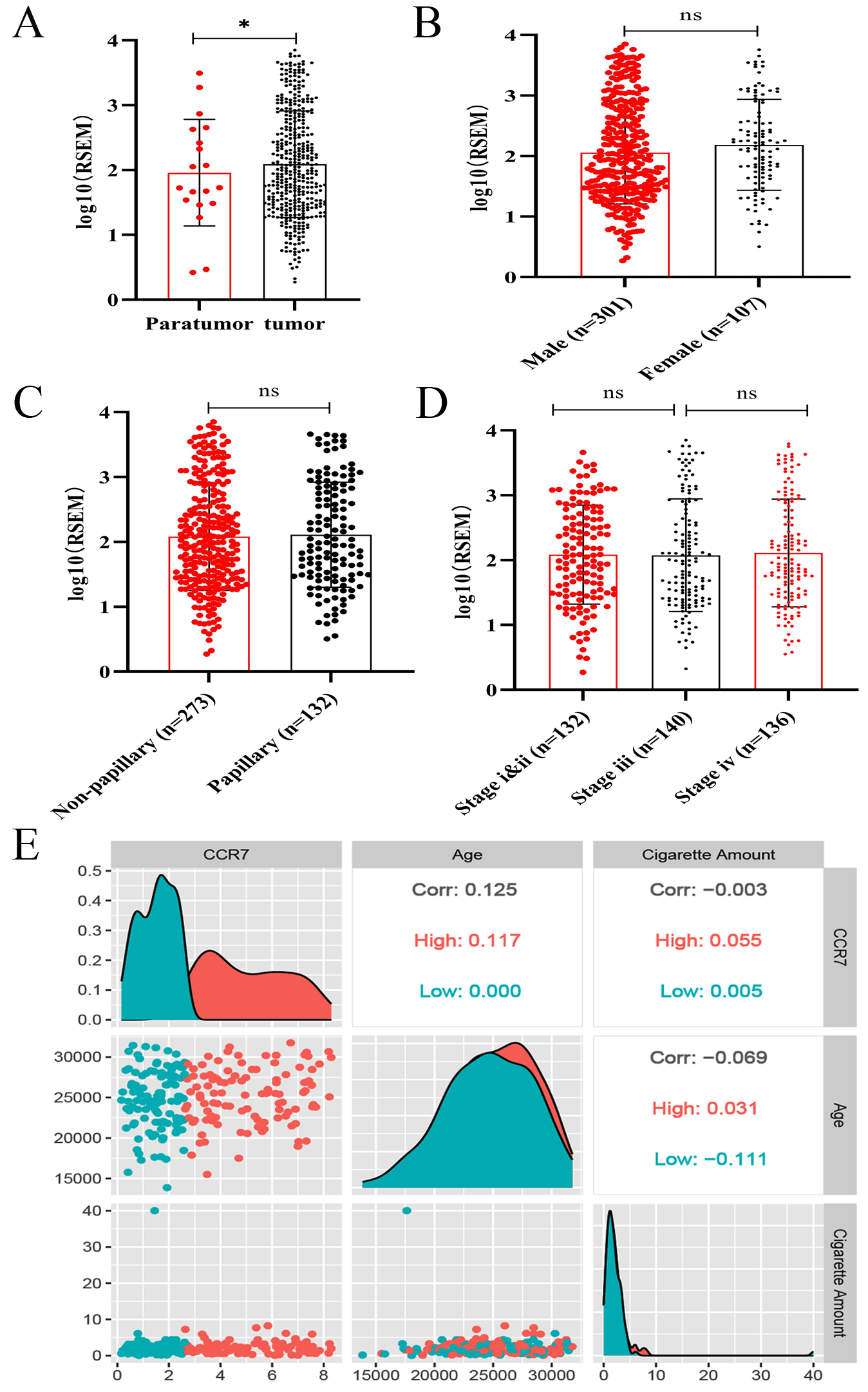

Supplement: Supplementary file 5 — Supplementary Material 5: Supplementary Figure 5. Comparative analysis of the expression in the TCGA dataset with clinically relevant tumour data. (A) Histogram plot showing the difference in CCR7 expression between paratumour and tumour bladder tissues. (B) Histogram plot showing the difference in CCR7 expression between bladder tissues of male and female patients. (C) Histogram plot showing the difference in CCR7 expression between different types of bladder tumour tissues.(D) Histogram plot showing the difference in CCR7 expression among different stages of bladder tumour tissues. (E) Correlation analysis of CCR7 high- and low-expression groups with clinical features in BLCA samples from the TCGA database. [file 12979_2024_432_MOESM5_ESM.tif]

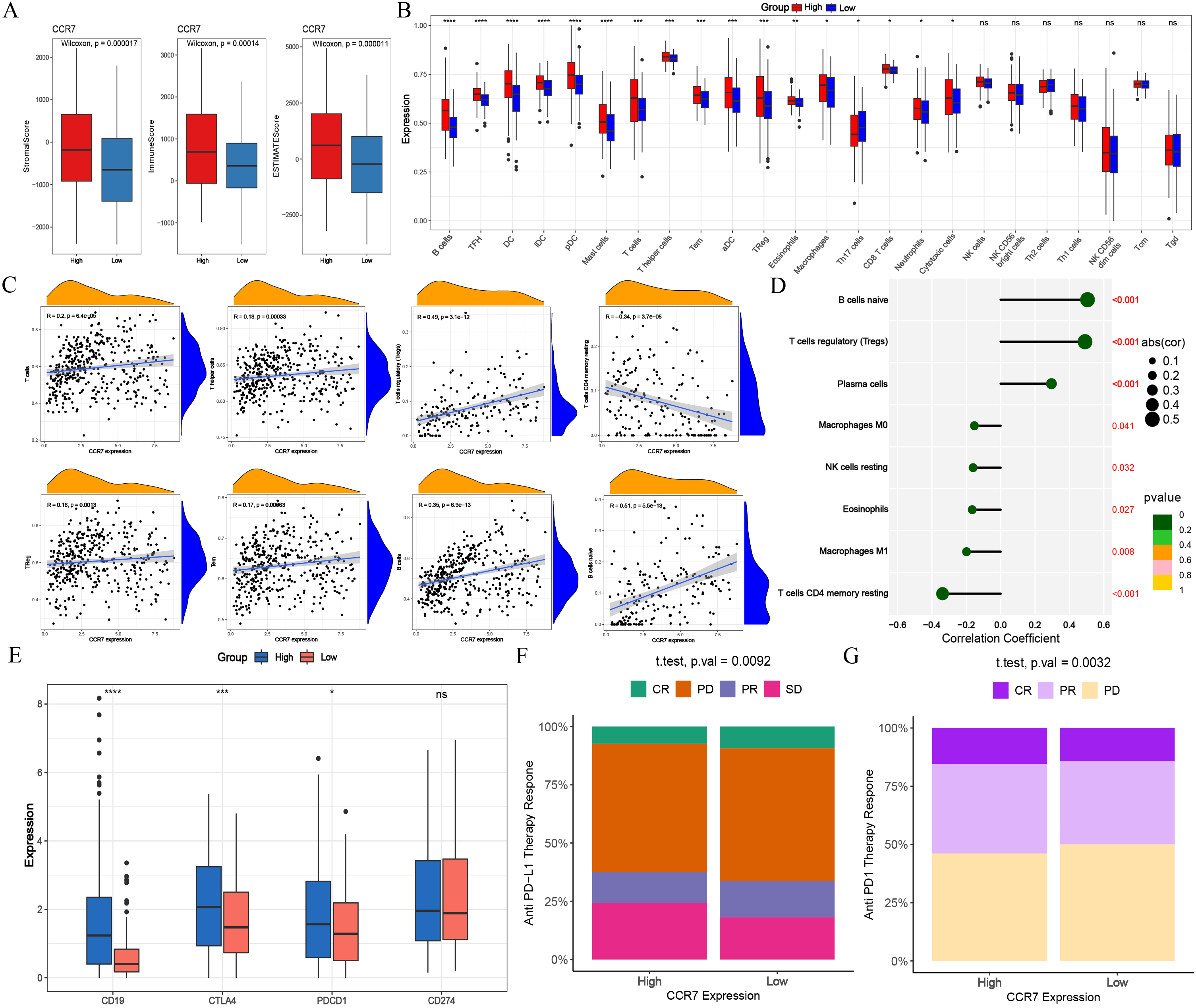

Supplement: Supplementary file 6 — Supplementary Material 6: Supplementary Figure 6. Correlation between CCR7 expression and immune infiltration and immunotherapy in bladder cancer. (A) Analysis of stromal, immune and ESTIMATE scores in the high and low CCR7 expression groups of bladder cancer. (B) ssGSEA analysis showing the distribution of immune cells in the high and low CCR7 expression groups. (C) Correlation analysis between CCR7 expression and T- and B-cell infiltration. (D) CIBERSORT analysis showing immune cell distribution. (E) Differential expression of immune checkpoint molecules between the high and low CCR7 expression groups. (F and G) Anti-PD-L1 and anti-PD-1 therapy response in the high and low CCR7 expression groups. * P < 0.05;** P < 0.01; *** P < 0.001; ns, not significant.(TIF 23629 KB) [file 12979_2024_432_MOESM6_ESM.tif]

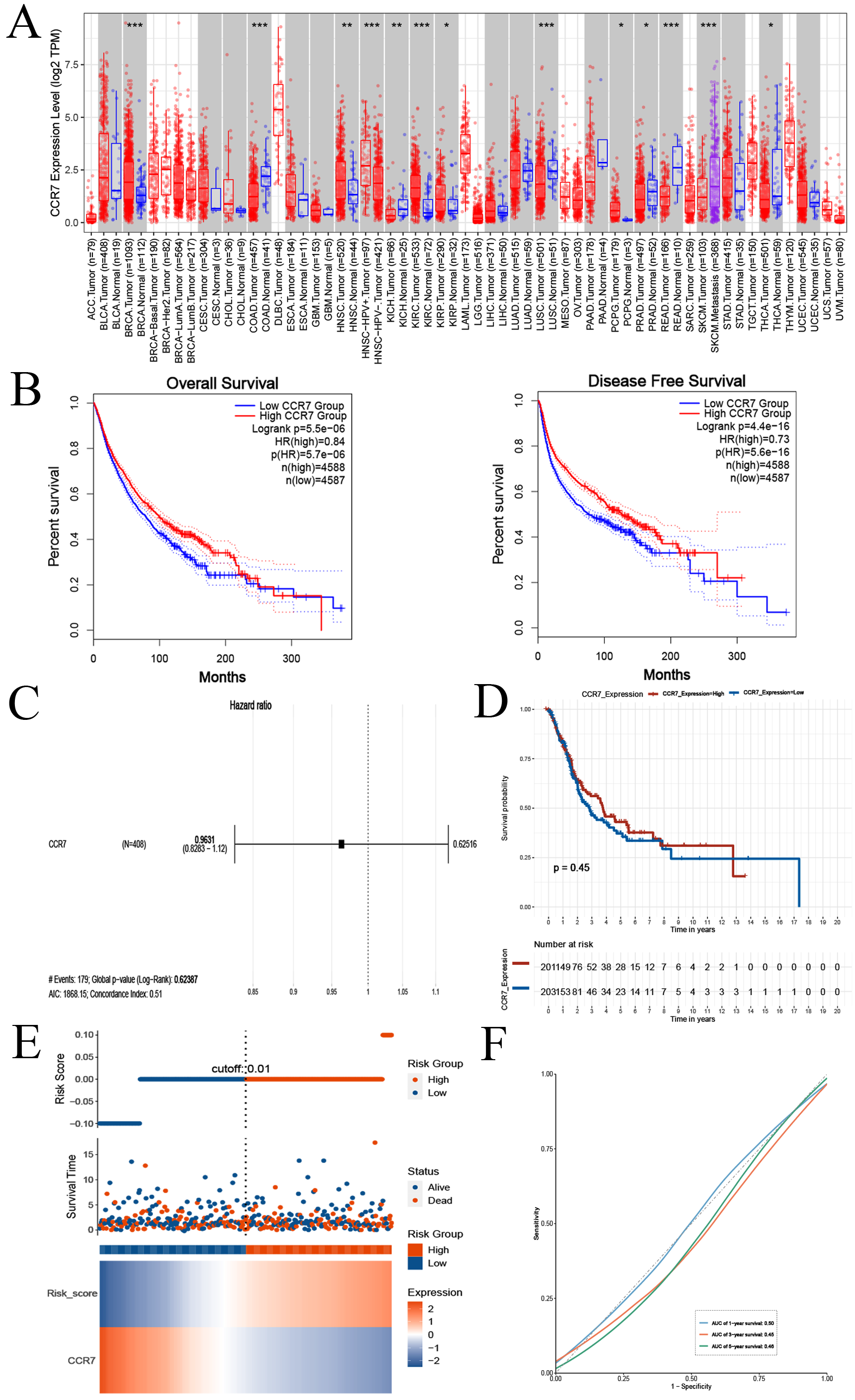

Supplement: Supplementary file 7 — Supplementary Material 7: Supplementary Figure 7. Prognostic analysis of CCR7 expression and bladder cancer. (A) Expression analysis of CCR7 in pan-cancer. (B) CCR7 expression in pan-cancer analysed with overall tumour survival and disease-free survival. (C) Forest plot analysis of the HR of CCR7 in patients with bladder tumour. (D) Kaplan–Meier survival curves of time for patients with high and low CCR7 expression in bladder cancer. (E) Survival state distribution in patients with bladder cancer between high and low CCR7 expression. (F) AUC showing the efficiency of CCR7 in predicting outcomes in patients with bladder cancer. [file 12979_2024_432_MOESM7_ESM.tif]
