# Supplementary material for: Chemokine receptor 7 contributes to T- and B-cell filtering in ageing bladder, cystitis and bladder cancer
Source: Immun Ageing. 2024 May 18;21:33. doi: 10.1186/s12979-024-00432-5 (PMC11102276; doi:10.1186/s12979-024-00432-5)
Supplement: Supplementary file 8 — Supplementary Material 8: Supplementary Table 1. Clinical features of the high and low CCR7 expression groups in the cell membrane. [file 12979_2024_432_MOESM8_ESM.docx]

Supplementary Table 1. Clinical features of the high and low CCR7 expression groups in the cell membrane.

| CCR7 cell membrane scores | | | | | | |
| --- | --- | --- | --- | --- | --- | --- |
|  |  | Low | High | Total | χ² | p |
| Sex | Male | 19 | 24 | 43 | 2.15 | 0.143 |
|  | Female | 7 | 2 | 9 |  |  |
| Age | <75 | 15 | 19 | 34 | 0.981 | 0.322 |
|  | ≥75 | 10 | 7 | 17 |  |  |
| Tumour size | <5 cm | 14 | 14 | 28 | 0 | 1 |
|  | ≥5 cm | 9 | 9 | 18 |  |  |
| T | Tis/T1/T2 | 7 | 16 | 23 | 7.671 | 0.006 |
|  | T3/T4 | 17 | 7 | 24 |  |  |
| TNM | Ois/1/2 | 5 | 11 | 16 | 4.454 | 0.035 |
|  | 3/4 | 18 | 10 | 28 |  |  |
| Grade | Low grade | 2 | 4 | 6 | 0.146 | 0.702 |
|  | High grade | 22 | 21 | 43 |  |  |
| Lymph node positivity | <1 | 14 | 14 | 28 | 0 | 1 |
|  | ≥1 | 3 | 4 | 7 |  |  |
| CD8 positivity rate | <5% | 9 | 19 | 28 | 7.738 | 0.005 |
|  | ≥5% | 17 | 7 | 24 |  |  |
| PDL-1 positivity rate | <5% | 9 | 15 | 24 | 2.786 | 0.095 |
|  | ≥5% | 17 | 11 | 28 |  |  |

Statistically significant (p < 0.05)
