# Supplementary material for: Chemokine receptor 7 contributes to T- and B-cell filtering in ageing bladder, cystitis and bladder cancer
Source: Immun Ageing. 2024 May 18;21:33. doi: 10.1186/s12979-024-00432-5 (PMC11102276; doi:10.1186/s12979-024-00432-5)
Supplement: Supplementary file 9 — Supplementary Material 9: Supplementary Table 2. Clinical features of the high and low CCR7 expression groups in the cytoplasm. [file 12979_2024_432_MOESM9_ESM.docx]

Supplementary Table 2. Clinical features of the high and low CCR7 expression groups in the cytoplasm.

| CCR7 cytoplasmic scores | | | | | | |
| --- | --- | --- | --- | --- | --- | --- |
|  |  | Low | High | Total | χ² | p |
| Sex | Male | 22 | 21 | 43 | 0 | 1 |
|  | Female | 4 | 5 | 9 |  |  |
| Age | <75 | 20 | 14 | 34 | 2.511 | 0.113 |
|  | ≥75 | 6 | 11 | 17 |  |  |
| Tumour size | <5 cm | 14 | 14 | 28 | 0 | 1 |
|  | ≥5 cm | 9 | 9 | 18 |  |  |
| T | Tis/T1/T2 | 7 | 16 | 23 | 6.17 | 0.013 |
|  | T3/T4 | 16 | 8 | 24 |  |  |
| TNM | Ois/1/2 | 4 | 12 | 16 | 5.877 | 0.015 |
|  | 3/4 | 19 | 9 | 28 |  |  |
| Grade | Low grade | 3 | 3 | 6 | 0 | 1 |
|  | High grade | 22 | 21 | 43 |  |  |
| Lymph node positivity | <1 | 13 | 15 | 28 | 3.483 | 0.062 |
|  | ≥1 | 6 | 1 | 7 |  |  |
| CD8 positivity rate | <5% | 12 | 16 | 28 | 1.238 | 0.266 |
|  | ≥5% | 14 | 10 | 24 |  |  |
| PDL-1 positivity rate | <5% | 12 | 12 | 24 | 0 | 1 |
|  | ≥5% | 14 | 14 | 28 |  |  |

Statistically significant (p < 0.05)
