# Supplementary material for: Chemokine receptor 7 contributes to T- and B-cell filtering in ageing bladder, cystitis and bladder cancer
Source: Immun Ageing. 2024 May 18;21:33. doi: 10.1186/s12979-024-00432-5 (PMC11102276; doi:10.1186/s12979-024-00432-5)
Supplement: Supplementary file 10 — Supplementary Material 10: Supplementary Table 3. Cox regression analysis. [file 12979_2024_432_MOESM10_ESM.docx]

Supplementary Table 3. Cox regression analysis.

|  | Univariate analysis | | | | Multivariate analysis | | | |
| --- | --- | --- | --- | --- | --- | --- | --- | --- |
|  | HR | 95% CI | | p value | HR | 95% CI | | p value |
|  |  | lower-bound | upper-bound |  |  | lower-bound | upper-bound |  |
| CCR7 cytoplasm score | 0.871 | 0.423 | 1.794 | 0.709 |  |  |  |  |
| CCR7 cell membrane score | 2.196 | 1.042 | 4.628 | 0.039 | 4.166 | 1.399 | 12.406 | 0.01 |
| Sex | 0.786 | 0.319 | 1.937 | 0.601 |  |  |  |  |
| Age | 0.72 | 0.339 | 1.527 | 0.392 |  |  |  |  |
| Tumour size | 1.47 | 0.66 | 3.272 | 0.346 |  |  |  |  |
| T | 0.526 | 0.25 | 1.106 | 0.090 |  |  |  |  |
| TNM | 0.337 | 0.133 | 0.855 | 0.022 | 0.943 | 0.304 | 2.923 | 0.919 |
| grade | 0.512 | 0.121 | 2.166 | 0.363 |  |  |  |  |
| Lymph node positivity | 0.34 | 0.118 | 0.979 | 0.045 | 0.197 | 0.053 | 0.738 | 0.016 |
| CD8 positivity rate | 1.58 | 0.743 | 3.363 | 0.235 |  |  |  |  |
| PDL-1 positivity rate | 0.897 | 0.437 | 1.839 | 0.766 |  |  |  |  |

* Statistically significant (p < 0.05)
