# Supplementary material for: Chemokine receptor 7 contributes to T- and B-cell filtering in ageing bladder, cystitis and bladder cancer
Source: Immun Ageing. 2024 May 18;21:33. doi: 10.1186/s12979-024-00432-5 (PMC11102276; doi:10.1186/s12979-024-00432-5)
Supplement: Supplementary file 11 — Supplementary Material 11: Supplementary Table 4. Quantitative real-time PCR. [file 12979_2024_432_MOESM11_ESM.docx]

Supplementary Table 4. Quantitative real-time PCR.

| Primer name | Sequence (5ʹ–3ʹ) |
| --- | --- |
| Human CCR7-F | TGAGGTCACGGACGATTACAT |
| Human CCR7-R | GTAGGCCCACGAAACAAATGAT |
| Mouse CCR7-F | GATGACTACATCGGCGAGAATA |
| Mouse CCR7-R | ACGAAGCAGATGACAGAATACA |
| β-actin-F | CAACGAGCGGTTCAGGTGT |
| β-actin-R | TGGAGTTGAAGGTGGTCTCG |
